# Circuit quantum electrodynamics detection of induced two-fold anisotropic pairing in a hybrid superconductor–ferromagnet bilayer

**DOI:** 10.1038/s41567-024-02613-x

**Published:** 2024-08-12

**Authors:** C. G. L. Bøttcher, N. R. Poniatowski, A. Grankin, M. E. Wesson, Z. Yan, U. Vool, V. M. Galitski, A. Yacoby

**Affiliations:** 1 Department of Physics, Harvard University, Cambridge, MA USA; 2 Joint Quantum Institute, Department of Physics, University of Maryland, College Park, MD USA; 3 Harvard John A. Paulson School of Engineering and Applied Sciences, Harvard University, Cambridge, MA USA; 4 Max Planck Institute for Chemical Physics of Solids, Dresden, Germany; 5 Center for Computational Quantum Physics, The Flatiron Institute, New York, NY USA; 6 Present Address: Department of Applied Physics, Yale University, New Haven, CT USA

**Keywords:** Superconducting properties and materials, Quantum information, Ferromagnetism

## Abstract

Hybrid systems represent one of the frontiers in the study of unconventional superconductivity and are a promising platform to realize topological superconducting states. These materials are challenging to probe using many conventional measurement techniques because of their mesoscopic dimensions, and therefore require new experimental probes so that they can be successfully characterized. Here, we demonstrate a probe that enables us to measure the superfluid density of micrometre-size superconductors using microwave techniques drawn from circuit quantum electrodynamics. We apply this technique to a superconductor–ferromagnet bilayer and find that the proximity-induced superfluid density is two-fold anisotropic within the plane of the sample. It also exhibits power-law temperature scaling that is indicative of a nodal superconducting state. These experimental results are consistent with the theoretically predicted signatures of induced triplet pairing with a nodal *p*-wave order parameter. Moreover, we observe modifications to the microwave response at frequencies near the ferromagnetic resonance, suggesting a coupling between the spin dynamics and induced superconducting order in the ferromagnetic layer. Our experimental technique can be employed more widely, for example to study fragile unconventional superconductivity in low-dimensional materials such as van der Waals heterostructures.

## Abstract (web summary)

Heterostructures of ferromagnets and superconductors may host exotic superconducting states. Now a circuit quantum electrodynamics technique is demonstrated that provides evidence for triplet *p*-wave pairing in such a heterostructure.

## Main

Heterostructures constructed from superconductors and other materials (for example, semiconductors, ferromagnets and topological materials) offer a rich platform to realize unconventional superconducting states via proximity effects. In these hybrid systems, the coupling between distinct materials leads to the formation of emergent phases that feature new physical properties that are otherwise absent in the isolated constituents. These include topological superconducting phases^1^ hosting non-Abelian excitations and states supporting spin-triplet pairing^2^, both of which have potential applications for quantum computing technology^3^. Given the extreme scarcity of naturally occurring topological^4–7^ or spin-triplet superconductors^8–11^, hybrid systems are an invaluable resource to realize these exotic superconducting states^12–15^.

The archetypal superconducting hybrid system is the superconductor–ferromagnet (S–F) bilayer, where spin-triplet superconductivity can be induced in the ferromagnet because of the combined effect of the exchange field and superconducting proximity effect^16,17^. While S–F heterostructures have been extensively studied using transport techniques, direct probes of the induced superconducting order have been lacking. This disparity lies in the fact that the mesoscopic nature of the induced superconducting state, which is tightly confined to the S–F interface and exists over nanometre-scale distances, renders most well-developed techniques in the study of bulk unconventional superconductors challenging to apply. Although there has been promising recent work applying conventional techniques to superconductor heterostructures^18–20^, new experimental probes are required to enable the direct study of induced unconventional superconducting states in hybrid superconducting systems^21,22^.

In this work, we employ an on-chip superconducting microwave resonator as a sensitive probe of a micrometre-scale S–F bilayer. Resonator circuits allow for the creation and control of highly localized electromagnetic fields, enabling one to attain strong coupling even to micrometre-scale samples. Consequently, superconducting resonators have already been widely developed as a powerful tool to study magnon dynamics^23–27^. By galvanically coupling a S–F bilayer to our resonator circuit, one can probe the inductive response of the bilayer, as has been employed to study superconductor–semiconductor systems^21,28^. This inductance is a direct manifestation of the induced superfluid density in the hybrid system.

When a metallic ferromagnet is placed into contact with a conventional *s*-wave superconductor, the strong exchange field in the ferromagnet de-pairs the spin-singlet Cooper pairs inherited from the superconductor and suppresses induced singlet superconductivity^29^. However, interfacial spin–orbit coupling (which is generically present) or magnetic inhomogeneities can flip the spin of an electron as it tunnels across the S–F interface and convert singlet pairs into spin-triplet Cooper pairs that can survive in the ferromagnet, leading to the formation of a mini-gap in the majority spin band in the ferromagnet^30,31^. To satisfy fermionic antisymmetry, these triplet pairs must either have an odd-parity (for example, *p*-wave) orbital structure or an odd-frequency pairing structure, with the pairing correlations being antisymmetric with respect to time^17,32^. Although the presence of triplet pairs has been indirectly inferred from the persistence of long-range supercurrents in long S–F–S Josephson junctions^33–35^, the detailed symmetry of the induced pairing is not yet well understood.

**Fig. 1:**
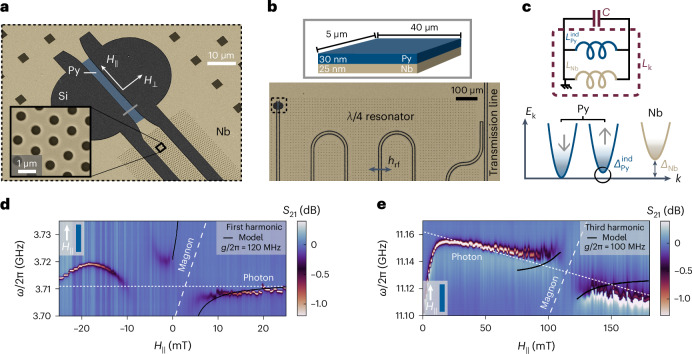
Device geometry and FMR. **a**,**b**, False-coloured scanning electron micrograph of the S–F bilayer (**a**), with the illustrated cross-section (**b**). The bilayer is integrated into a quarter-wavelength coplanar resonator patterned into the Nb film, shown in an optical micrograph in **b**. **c**, Top: at microwave frequencies, the bilayer response can be treated as a circuit of two parallel inductors, corresponding to the kinetic inductances associated with the bulk Nb superfluid density (${L}_{{{{\rm{Nb}}}}} \propto 1/{n}_{\mathrm{s}}^{{{{\rm{Nb}}}}}$) and the induced superfluid density in the bilayer. Bottom: as a result of their direct contact, the Nb is able to proximity-induce superconductivity in the Py stripe, leading to the formation of a mini-gap *Δ*_Py_ in the majority spin band. **d**, Transmission *S*_21_ across the circuit as a function of in-plane magnetic field *μ*_0_*H*_∥_ oriented along the length of the Py stripe. When the resonator frequency is tuned to the FMR frequency of the Kittel magnons in the Py, an anticrossing is observed in the resonator spectrum. The black lines are an overlay of the model spectral function (Supplementary Section VI) used to extract an effective magnon–photon coupling strength *g*/2π = 120 MHz. **e**, Transmission spectrum at the third harmonic of the resonator. Anticrossings are now observed at a higher field *μ*_0_*H*_∥_ ≈ 120 mT, where the FMR crosses the third harmonic frequency of ≈11 GHz. Fitting the transmission spectrum (black lines) yields a similar coupling *g*/2π = 100 MHz to that observed at the first harmonic. The broad shoulder on the left-hand side of both sweeps is due to hysteretic effects related to trapped flux in the superconducting resonator. λ, resonator wavelength; *h*_rf_, resonator rf magnetic field; *E*_k_, electronic energy; ind, induced.

To directly address the induced superconducting state in an S–F bilayer requires a probe that is amenable to the small (nanometre to micrometre scale) spatial size of typical devices, as well as the ability to selectively address the weak induced superconducting state that exists in parallel with the intrinsic bulk superconductivity of the superconducting layer. To achieve both of these requirements, we employ an on-chip superconducting coplanar waveguide resonator, which has been extensively developed as a part of the circuit quantum electrodynamics architecture for superconducting quantum information devices^36^. The resonator is fabricated from a 25-nm-thick Nb film perforated with flux pinning holes to maximize performance in external magnetic fields^37^, in a quarter-wavelength configuration with one end of the resonator shorted to ground and the other open (Fig. 1a,b). These Nb resonators are designed to have resonance frequencies *ω*_r_/2π = 4−7 GHz and attain quality factors of *Q* ≈ 350,000 at our base operating temperature of *T* ≈ 55 mK, enabling high sensitivity in our measurements.

To study the superconducting state of an S–F bilayer, we deposit a 30-nm-thick permalloy (Py) stripe directly on top of the Nb centre conductor where the resonator is shorted to ground, forming a Nb–Py bilayer that is situated at a current antinode of the circuit as shown in Fig. 1a. Because the current is concentrated at the location of the bilayer, the resonator response is dominated by the properties of the S–F subsystem. After the Py stripe is deposited onto the resonator, the resonator frequency shifts down by about 100 MHz relative to its bare value without Py, reflecting an increase in kinetic inductance originating from the S–F bilayer. Moreover, there is a drastic reduction in the quality factor of the Py-loaded resonator to *Q* ≈ 7,000, which already reflects a strong coupling between the magnetic and superconducting subsystems.

Moreover, spectroscopy of the hanger style resonator, interrogated via the transmission *S*_21_ across a capacitively coupled transmission line (Fig. 1b), allows one to directly probe the microwave dynamics of the ferromagnet. In particular, by applying an in-plane magnetic field *H*_∥_ along the length of the Py stripe, we may tune the ferromagnetic resonance (FMR) frequency, which follows the Kittel law ${\omega }_{{{{\rm{m}}}}}({H}_{\parallel })=\gamma \sqrt{({H}_{\parallel }+{M}_{\mathrm{s}}){H}_{\parallel }}$, where *γ* is the gyromagnetic constant and *μ*_0_*M*_s_ ≈ 1.2 T is the saturation magnetization of Py^38,39^,wherein *μ*_0_ is the vacuum permeability. When the FMR frequency is brought to coincide with the frequency of the resonator mode, we observe clear anticrossings in the resonator spectrum associated with the formation of magnon-polaritons. These anticrossings are observed at both low fields *μ*_0_*H*_∥_ ≈ 7 mT when the FMR intersects the fundamental frequency of the resonator, and also higher fields *μ*_0_*H*_∥_ ≈ 110 mT when the FMR crosses the third harmonic of the resonator (a quarter-wavelength resonator exhibits only odd harmonics), as shown in Fig. 1d,e. In both cases, we can fit the resonator spectrum and extract an effective coupling strength *g*/2π ≈ 100 MHz between the resonator and FMR mode (Supplementary Section VI). From the dimensions of the Py stripe, this corresponds to a coupling strength of 150 Hz per spin, which drastically exceeds the coupling strengths reported in previous works^26,27^ on Py–Nb hybrid circuits, where the Py stripe was separated from the superconductor with an insulating layer to prevent any degradation in *Q* from the inverse proximity effect. In contrast, our devices feature a direct interface between the superconductor and ferromagnet, enabling proximity effects and the possibility of interesting dynamics generated by the interplay between the order parameters in each layer of the hybrid S–F system.

At microwave frequencies, a superconductor behaves as an inductive element characterized by a kinetic inductance arising from the superfluid response^40^. This inductance is fundamentally related to the density *n*_s_ of superfluid carriers as *L*_k_ = (*m*/*n*_s_*e*^2^)(*ℓ*/*s*) for a superconducting wire of length *ℓ* and cross-section *s*, where *m* is the electron mass and *e* is the electron charge. Notably, the kinetic inductance is large for fragile or dilute superconductors with a small superfluid density, and for thin systems with small cross-sections. Both of these features make kinetic inductance measurements especially favourable for probing weak and low-dimensional superconductors, such as the proximity-induced superconducting state in an S–F bilayer. The kinetic inductance is reflected in the resonant frequency $2\uppi f=1/\sqrt{({L}_{{{{\rm{g}}}}}+{L}_{\mathrm{k}})C}$, where *L*_g_ and *C* are the geometric inductance and capacitance of the circuit, respectively. When the system is weakly perturbed by changing an external parameter such as the temperature or applied magnetic field (under the reasonable assumption that *L*_g_ and *C* are constant), the frequency shift of the resonator is directly proportional to the change in the kinetic inductance or, equivalently, in the superfluid density

1 $$\frac{\delta f}{{f}_{0}}\approx -\frac{1}{2}\frac{\delta {L}_{\mathrm{k}}}{{L}_{\mathrm{k},0}}\approx \frac{\kappa }{2}\frac{\delta {n}_{\mathrm{s}}}{{n}_{\mathrm{s},0}}\,,$$

where we have assumed that the frequency shift *δf* is small compared to the resonance frequency *f*_0_ at our base operating temperature of 55 mK such that *δf*/*f*_0_ ≪ 1, and have introduced the kinetic inductance fraction *κ* = *L*_k,0_/(*L*_g_ + *L*_k,0_). Thus, by studying the evolution of the resonator frequency with temperature or magnetic field, we can sensitively measure the changes in the superfluid density of the S–F bilayer, offering a direct probe of the induced superconducting order.

In fact, superfluid density measurements have proven to be an essential tool in the study of bulk unconventional superconductivity^41,42^. In conventional fully gapped superconductors, the superfluid density exhibits a thermally activated temperature (*T*) dependence $\delta {n}_{\mathrm{s}}(T\;)/{n}_{\mathrm{s},0}\equiv \left[{n}_{\mathrm{s}}(T\;)-{n}_{\mathrm{s}}(0)\right]/{n}_{\mathrm{s}}(0)\propto {{{{\rm{e}}}}}^{-{{\varDelta }}/T}/\sqrt{T}$, where *Δ* is the superconducting energy gap. In contrast, unconventional superconductors with nodal order parameters host low-lying quasiparticles residing at the gap nodes, leading to a power-law dependence of the superfluid density *δn*_s_(*T*)/*n*_s,0_ ∝ *T*^*n*^, where the exponent *n* depends on spatial dimensionality, the dimensionality of the nodes and the degree of disorder in the system^41,42^.

**Fig. 2:**
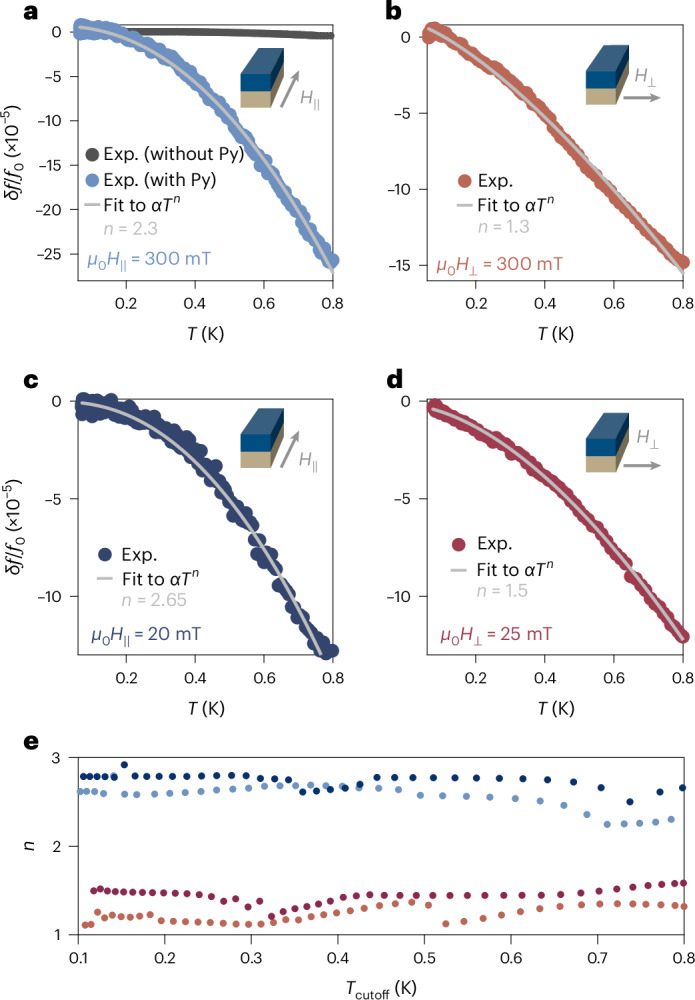
Anisotropic temperature dependence of inductance. **a**, Shift in resonance frequency *δf*/*f*_0_ = [*f*(*T*, *H*) − *f*(55 mK, *H*)]/*f*(55 mK, *H*) in an in-plane field *μ*_0_*H*_∥_ = 300 mT oriented along the length of the Py stripe, as illustrated in the inset. The comparatively negligible temperature dependence of the resonance frequency of a bare Nb resonator (without a Py stripe) is shown for comparison. **b**, Shift in the resonance frequency in an in-plane field *μ*_0_*H*_⊥_ = 300 mT oriented perpendicular to the length of the Py stripe. **c**, Frequency shift in an in-plane field *μ*_0_*H*_∥_ = 20 mT. **d**, Frequency shift in an in-plane field *μ*_0_*H*_⊥_ = 25 mT. In all plots, the grey line is a fit of the data over the full temperature range to the power-law dependence *δf*/*f*_0_ = *αT*^*n*^, with *α* and *n* as fitting parameters. **e**, Extracted temperature-scaling exponent *n* as a function of the upper cutoff of the temperature range over which the data are fit, for the data in each panel **b**–**d**. Irrespective of the details of the fit procedure, the scaling exponents for fields parallel and perpendicular to the stripe are distinct. Exp., experiment. Source data

We may simplistically imagine that the microwave response of the bilayer can be described as that of two parallel inductors, as illustrated in Fig. 1c: one corresponding to the kinetic inductance of the induced superconducting state in the Py, and the other corresponding to the bulk superfluid density of the Nb film below. We will focus on the low-temperature regime *T* ≲ 800 mK in our measurements, well below the critical temperature ${T}_{\rm{c}}^{{{\;{\rm{Nb}}}}}\approx 8\,{\mathrm{K}}$, such that the kinetic inductance of the Nb film is effectively frozen out and equal to its zero-temperature value. Experimentally, as shown in Fig. 2a, the resonance frequency of bare Nb resonators exhibits very little temperature dependence in this range, with *δf*/*f*_0_ ≈ 10^−6^, consistent with this assumption. We have further validated this technique by measuring the superfluid density of a small micrometre-scale Al film inserted at the end of the resonator (Supplementary Section V), which leads to an activated temperature dependence of the resonance frequency with a rate consistent with the gap of Al. Thus, we can attribute the temperature-dependent changes studied below to the microwave response of the S–F bilayer.

We may begin by studying the response of the hybrid resonator in an applied in-plane magnetic field so that the FMR is detuned to be far above our operating frequency, which in this case is *ω*_r_/2π ≈ 7 GHz. The system’s behaviour when the FMR is near the resonator frequency, and the cavity mode takes on the character of a magnon-polariton, will be discussed later. In Fig. 2a, we present the temperature dependence of the fundamental resonant frequency of the hybrid S–F circuit in an in-plane magnetic field of *μ*_0_*H*_∥_ = 300 mT oriented along the length of the Py stripe, parallel to the direction of the microwave current (see inset). Notably, the temperature dependence is manifestly non-exponential, in contrast to the expectation for a conventional fully gapped superconductor. Fitting the temperature-dependent frequency shift to a simple power law, *δf*/*f*_0_ = *αT*^*n*^, we find an exponent of *n* = 2.3. The overall magnitude *α* of the frequency shift is determined by several non-universal factors, and we will primarily focus on the exponent *n* throughout this work (see Supplementary Section VIII for further discussion).

In Fig. 2b, we instead apply the field perpendicular to the current and present the temperature dependence of *δf*/*f*_0_ at *μ*_0_*H*_⊥_ = 300 mT. We again find a power-law, rather than exponential, temperature dependence with a different, faster exponent *n* = 1.3 compared to the *H*_∥_ configuration. That is, we observe a two-fold anisotropy in the temperature scaling of the hybrid resonator frequency, and by extension of the kinetic inductance of the S–F bilayer.

We may also perform measurements at lower magnetic fields and probe the temperature dependence of the resonance below the FMR frequency. In Fig. 2c,d we present *δf*/*f*_0_ traces for *μ*_0_*H*_∥_ = 20 mT and *μ*_0_*H*_⊥_ = 25 mT, where we again see power-law temperature dependences in both cases. Further, we again find a two-fold anisotropy in the exponent *n*, with *n* = 2.65 in the parallel configuration and *n* = 1.5 in the perpendicular configuration. Thus, we find that the temperature-dependent response of the S–F bilayer is qualitatively unchanged by the magnitude of the applied magnetic field.

The temperature in our dilution refrigerator is only stable below 800 mK, constraining the accessible temperature range for our measurements. To ensure that our results are independent of this upper limit, we may restrict the fits of the temperature-dependent resonance frequencies to progressively lower temperatures, and extract the scaling exponents *n* for each field orientation for different values of the upper cutoff of the fitting range, *T*_cutoff_. We plot the extracted exponents *n* as a function of *T*_cutoff_ in Fig. 2e, where we see that the scaling exponents for parallel versus perpendicular field orientations are clearly distinct independent of the fitting range, emphasizing the robustness of the observed scaling anisotropy.

It is natural to attribute the temperature dependence of the induced superfluid density, manifested in the shift of the hybrid S–F resonance, to the thermal excitations above the proximity-induced mini-gap. In particular, we note that the features we observe occur on temperature scales on the order of tens to hundreds of millikelvin, which is substantially smaller than the energy scales associated with either the superconductor (with a critical temperature of 8 K) or ferromagnet (with a Curie temperature of approximately 500 K) independently. This strongly suggests that the physics underlying the observed temperature-dependent response arises because of the low-energy coupling between the two states, for example from a proximity-induced superconducting state.

In general, a variety of superconducting correlations with different spin and orbital symmetries are generated at the S–F interface^16,17,43^. Typically, however, only the correlations that can persist over long distances (such as the odd-frequency triplet state) into the ferromagnet contribute meaningfully in traditional transport experiments, and hence have been the principal focus of theoretical study. Nonetheless, other superconducting correlations are always present, albeit potentially confined to the interface over atomic-scale distances and thus challenging to detect using conventional probes.

Our observation of a power-law, rather than activated, temperature dependence of the superfluid density suggests that we are coupling to a nodal, rather than fully gapped, induced superconducting state. Such an anisotropic state would not be protected by Anderson’s theorem and thus be susceptible to pair-breaking from impurity scattering, and consequently would be confined to within a mean free path of the S–F interface. The possibility of our experiment to detect such a weak state lies in the fact that we measure changes in the kinetic inductance, and thus are primarily sensitive to the lowest-lying thermally excited quasiparticles and the most fragile superconducting states, as opposed to being immediately shunted by the fully gapped superconducting state. Moreover, the lateral geometry of the bilayer integrated into our superconducting circuit enables even states localized to the S–F interface to contribute to the inductive response.

In general, the superfluid density is a tensor quantity that can have two distinct components in a (quasi-) two-dimensional system^41,44,45^. However, neither magnetostatic effects, for example stray fields, nor simple pair-breaking considerations can account for anisotropic temperature scaling of the superfluid density (Supplementary Section IX). In contrast, the superfluid density in a nodal superconductor can display different temperature scalings depending on the relative orientation between the current and nodal direction^45^. Intuitively, one can imagine that the gapless quasiparticle states residing near the gap nodes are most efficiently excited when the current is aligned along the nodal direction, leading to a temperature scaling *δn*_s_ ∝ *T* that reflects the linear dispersion of the nodal quasiparticles. In contrast, when the current is aligned along the antinodal direction, nodal quasiparticles are less efficiently excited, leading to a slower temperature dependence *δn*_s_ ∝ *T*^*n*^ with *n* > 1. In this case, the precise power-law dependence of the superfluid density is determined by the microscopic details of the system (for example, spatial dimensionality, codimension of the gap nodes, disorder and so on).

Thus, our finding of a two-fold anisotropic power-law scaling of the superfluid density strongly constrains the possible superconducting states detected in our experiment. In particular, a two-fold anisotropy is only consistent with an induced order parameter with a *p*-wave orbital symmetry. Moreover, the power-law dependence of the superfluid density implies that the induced state is nodal, and that applying a d.c. magnetic field parallel or perpendicular to the microwave current allows one to selectively address the nodal or antinodal orientation of the *p*-wave order parameter.

To inform our experimental findings, we can construct a phenomenological model for the induced superfluid density in the S–F bilayer. We consider a bilayer system consisting of an *s*-wave superconductor and a ferromagnet with an in-plane magnetization oriented along the exchange field **H**_ex_. The interlayer tunnelling is assumed to have a spin-independent component, *t*, as well a component with the Rashba spin–orbit texture of the form ${t}_{{{{\rm{soc}}}}}{\left({{{\bf{k}}}}\times \sigma \right)}_{z}$ arising from the inversion symmetry breaking at the interface^31^. Here, *σ* is the electron spin and **k** is the in-plane electron momentum. We take the Zeeman field to lie in an in-plane orientation, which gives rise to a *p*-wave order parameter for the majority spin component of the ferromagnet, with the form ${{{\varDelta }}}_{{{{\bf{k}}}}}={{{\varDelta }}}_{\mathrm{t}}\cos \theta$, where *θ* is the angle between **k** and **H**_ex_, and *Δ*_t_ is the amplitude of the triplet order parameter (Supplementary Section I). Within the mean-field approximation, the Meissner kernel at temperature *T* is^46^

2 $$\delta {K}_{i,\;j}=\frac{-2{e}^{2}}{c}\int\limits_{0}^{\infty }{\mathrm{d}}\epsilon\, {n}_{\mathrm{F}}\left(\epsilon \right){\left\langle{\mathrm{Re}} \frac{{{{{\bf{v}}}}}_{i}{{{{\bf{v}}}}}_{j}{{{\varDelta }}}_{{{{\bf{k}}}}}^{2}}{{\left[{\left(\epsilon -{{\varSigma }}\right)}^{2}-{{{\varDelta }}}_{{{{\bf{k}}}}}^{2}\right]}^{3/2}}\right\rangle }_{\!\mathrm{FS}},$$

where 〈…〉_FS_ denotes a Fermi surface average; **v**_*i*_ is the Fermi velocity; ${n}_{\mathrm{F}}\left(\epsilon \right)$ is the Fermi distribution; $\delta {K}_{i,\;j}={K}_{i,\;j}\left(T\;\right)-{K}_{i,\;j}\left(0\right)$, where *i* and *j* are spatial indices; *c* is the speed of light; Re is for real; and ${{\varSigma }}\left(\epsilon \right)$ is the diagonal component of the self-energy, which we evaluate within the strong-scattering self-consistent *T-*matrix approximation ${\hat{\Sigma}}\left(\epsilon \right)={\tau}^{-1}/{\sum}_{k}{\hat{G}}_{k}$, where *τ* is the scattering time and $\hat{G}$ is the Nambu-electron Green’s function.

**Fig. 3:**
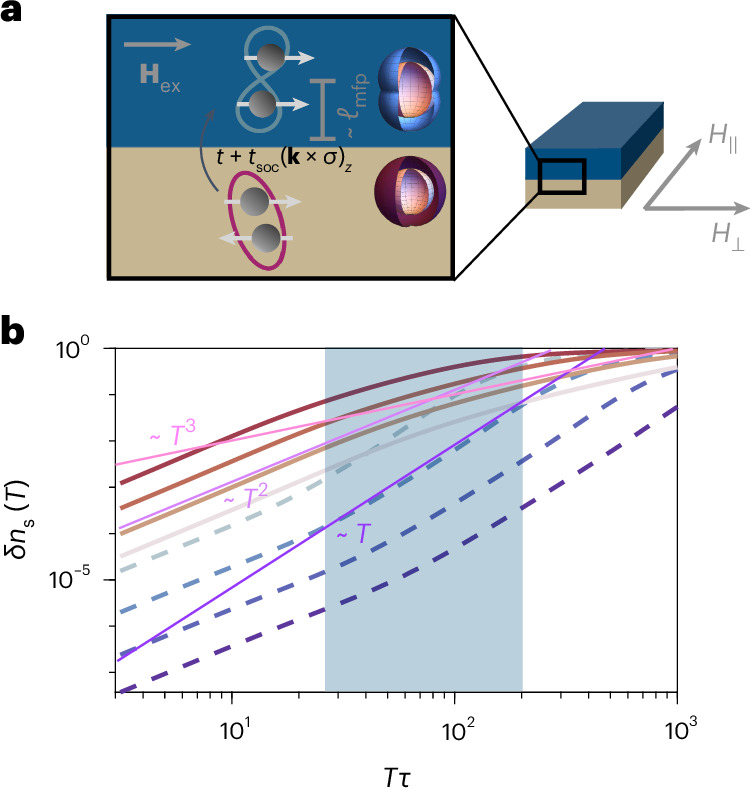
Superfluid density for a disorder nodal *p*-wave state. **a**, Illustration of the S–F bilayer with the in-plane field directions *H*_∥_, *H*_⊥_ indicated. The cross-section schematically depicts how interfacial spin–orbit coupling can convert isotropic spin-singlet pairs in the Nb layer into spin-triplet *p*-wave pairs in the ferromagnet. ℓ_mfp_, mean free path. **b**, Superfluid density $\delta {n}_{\mathrm{s}}\left(T\;\right)={n}_{\mathrm{s}}\left(T\;\right)-{n}_{\mathrm{s}}\left(T=0\right)$ as a function of temperature for Δ*τ* ≈ 5 × 10^2^, Δ*τ* ≈ 10^3^, Δ*τ* ≈ 3 × 10^3^, Δ*τ* ≈ 6 × 10^3^, where Δ is the triplet gap. Solid and dashed curves correspond to the response along and transverse to the nodes of the superconducting gap, respectively, where darker colours correspond to higher *τ*. Lines corresponding to temperature scalings of *T*, *T*^2^ and *T*^3^ are included in purple and pink as guides to the eye. The blue-shaded region indicates the range of parameter space compatible with the experimental results. Source data

In the clean limit, the low-temperature Meissner kernel scales as *T* and *T*^3^ when probed along the nodal and antinodal directions of the superconducting order parameter, respectively. Meanwhile, for strong disorder the low-temperature scaling is quadratic *δK*_*i*,*j*_ ∝ *T*^2^ in both directions. For a general temperature and disorder scattering time *τ*, the Meissner response can be evaluated numerically. The result for the anisotropic superfluid density defined as *n*_s_ = *K*_*i*,*j*_*c*/*e*^2^ is shown in Fig. 3, where we find that the temperature scaling continuously evolves from a quasi-isotropic *T*^2^ dependence at strong disorder to a strongly anisotropic *T* and *T*^3^ dependence along the nodal and antinodal directions, respectively, in the clean or high-temperature limit. Notably, the experimentally observed temperature scaling along the two directions is compatible with this theory over a wide region of parameter space, highlighted in blue in Fig. 3. However, the scattering rate in this region of parameter space is substantially smaller than the bare scattering rate we expect to be relevant for our Py films. That is, the clean limit of the theory qualitatively captures our observations, even though our samples are unquestionably dirty. The reason for this is an outstanding theoretical question, but we speculate that this could be because the induced superconductivity is confined to the S–F boundary over atomic length scales and thus experiences an effective scattering rate that is much smaller than the bare scattering rate in the bulk of the Py film. From a more technical perspective, our toy model cannot resolve atomic-scale features, and thus is not quantitatively accurate in capturing the fine structure of induced pairing at the interface. A more sophisticated quasiclassical approach to this problem, fully incorporating the role of disorder, is discussed at length in Supplementary Section II.

To intuitively understand the origin of these power laws, we recall that in a clean superconductor with line nodes one expects that the component of the superfluid density along the nodal direction scales linearly with temperature, reflecting the linear dispersion of the low-lying quasiparticles as discussed above. However, the introduction of weak non-magnetic disorder gives rise to low-lying impurity states that fill in the node, leading to a finite quasiparticle density of states at low energies, manifested as a quadratic temperature dependence of the superfluid density at low temperatures^46^. Above the energy scale *T*^⋆^ of these impurity states (which is set by the superconducting gap and impurity scattering rate), the usual linear-in-temperature scaling is recovered. In fact, such a quadratic-to-linear crossover has been extensively used to successfully describe superfluid density measurements of cuprate superconductors with varying degrees of disorder. In the language of temperature-scaling exponents, this quadratic-to-linear crossover translates to intermediate scaling exponents 1 < *n* < 2 in the nodal direction (as observed experimentally), where the precise value of *n* varies continuously with the degree of disorder. Similarly, one expects 2 < *n* < 3 in the antinodal direction, which is again consistent with the experimental results.

**Fig. 4:**
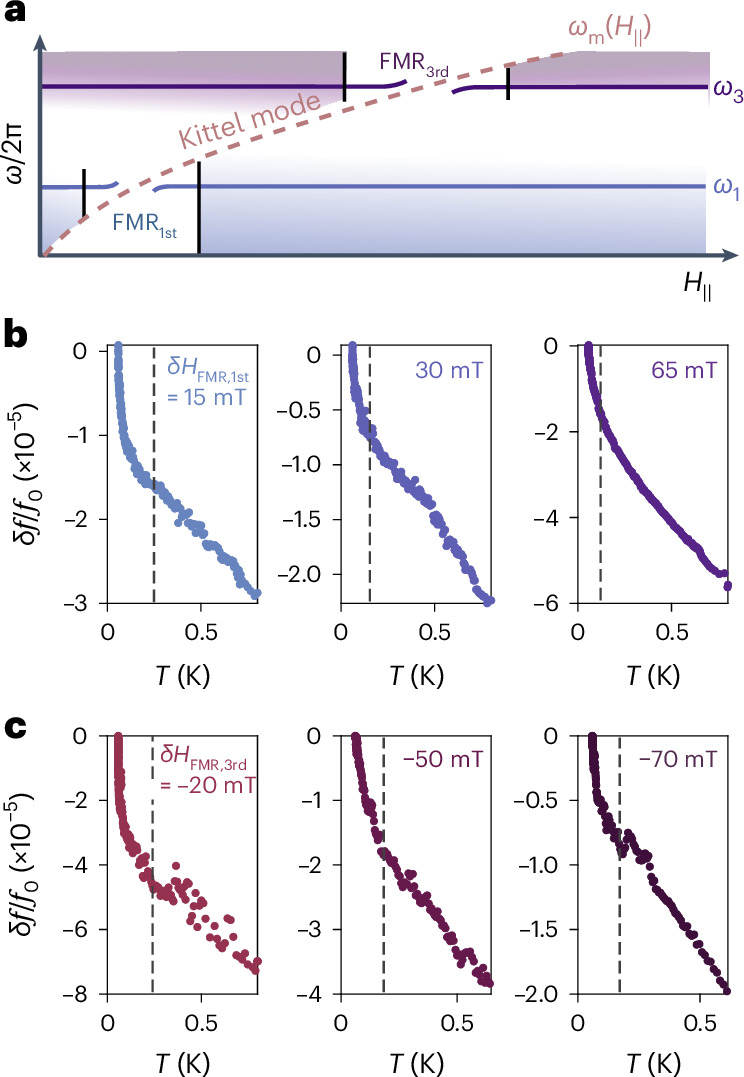
Temperature scaling near the FMR. **a**, Schematic illustration of the first and third harmonic modes of the resonator and the evolution of the FMR (Kittel) mode frequency with in-plane magnetic field. **b**, Temperature dependence of the first harmonic resonator frequency at fields above the FMR field, that is, *δH*_FMR,1st_ = *H* − *H*_FMR,1st_ > 0. Progressively steeper upturns in the temperature dependence are observed as the FMR is approached. **c**, Temperature dependence of the third harmonic resonator frequency at fields below the FMR field, *δH*_FMR,3rd_ < 0. The steepness of the upturns again scales with proximity to the FMR field. Dashed lines are guides to the eye that mark the approximate temperature at which the upturns onset. Source data

So far, we have focused on the temperature-dependent response of the hybrid S–F resonator subjected to in-plane magnetic fields such that the resonator is far detuned from the FMR frequency. If we perform the same measurements at fields where the resonator frequency is near the FMR frequency, we observe strikingly different behaviour as illustrated in Fig. 4. Namely, we observe a sharp upturn in the resonance frequency as the temperature is lowered, which can be described as a nearly divergent power law scaling as *δf*/*f*_0_ ∝ *T*^*n*^ with *n* < 1 at low temperatures. By comparing the response of the first and third harmonics, which intersect the FMR at different magnetic fields, we can confirm that these upturn features track with the proximity to the FMR field (that is, the field *H*_FMR_ such that *ω*_m_(*H*_FMR_) = *ω*_r_, where *ω*_m_ is the ferromagnetic resonance freqeuncy) as opposed to the magnitude of the in-plane magnetic field itself. These upturns become increasingly sharp as the FMR field is approached, and weaker upturns persist over a relatively wide field range, of the order of 100 mT, away from the FMR field. The exact field range over which the upturns persist is device dependent, but in all cases the upturns track with the FMR frequency.

The appearance of these low-temperature upturns, which manifest on temperature scales far lower than the relevant scales in either the superconductor or ferromagnet independently, are again indicative of strong interactions and hybridization between the two subsystems. However, on account of the unusual, seemingly divergent, temperature-scaling exponent *n* in this regime, it is unclear whether the temperature dependence of the resonance frequency near the FMR can be simply attributed to changes in the superfluid density of the S–F bilayer. We also note that qualitatively similar upturns, and history-dependent artefacts presumably related to trapped magnetic flux, are occasionally observed in the temperature dependence of bare Nb resonators after repeated magnetic field cycling (as elaborated on in Supplementary Section VII). In contrast, the upturns observed in the S–F devices near the FMR are a reproducible feature of the phenomenology of these devices.

Altogether, our kinetic inductance technique can sensitively detect fragile subdominant induced superconducting orders. Our work thus establishes kinetic inductance techniques as a complementary probe to conventional transport experiments in the study of hybrid superconducting devices, enabling a deeper understanding of induced unconventional superconductivity in these systems. More broadly, our technique is well suited to van der Waals superconductors^47^, where the nature of superconductivity remains poorly understood and, much like hybrid systems, the low dimensionality inhibits the use of many conventional probes. Moreover, the kinetic inductance in these materials is expected to be extremely large on account of their dilute carrier densities, resulting in larger responses and possible device functionalities. This technique is thus poised to lead to advances in the understanding of both induced superconductivity in hybrid systems and intrinsic superconductivity in two-dimensional materials.

## Online content

Any methods, additional references, Nature Portfolio reporting summaries, source data, extended data, supplementary information, acknowledgements, peer review information; details of author contributions and competing interests; and statements of data and code availability are available at 10.1038/s41567-024-02613-x.

## Supplementary information

Supplementary Information Supplementary Sections I–IX and Figs. 1–6.

## Source data

Source Data Fig. 2 Data for Fig. 2a–d.

Source Data Fig. 3 Data for Fig. 3b.

Source Data Fig. 4 Data for Fig. 4b,c.

## Data Availability

The data supporting the findings of this study are available upon request. Source data are provided with this paper.
