# Supplementary material for: Circuit quantum electrodynamics detection of induced two-fold anisotropic pairing in a hybrid superconductor–ferromagnet bilayer
Source: Nat Phys. 2024 Aug 12;20(10):1609–15. doi: 10.1038/s41567-024-02613-x (PMC11473361; doi:10.1038/s41567-024-02613-x)
Supplement: Supplementary file 1 — Supplementary Sections I–IX and Figs. 1–6. [file 41567_2024_2613_MOESM1_ESM.pdf]

nature physics

Supplementary information

https://doi.org/10.1038/s41567-024-02613-x

Circuit quantum electrodynamics detection of induced two-fold anisotropic pairing in a hybrid superconductor–ferromagnet bilayer

In the format provided by the authors and unedited

CONTENTS

I. Technical details of the calculation of the superfluid density 1
A. Self-energy and vertex corrections 1

II. Quasiclassical approach 2
A. Numerical solution 2
B. Generation and propagation of p-wave triplet 2

III. Device fabrication 4

IV. Experimental setup 4

V. Measuring the superconducting gap of aluminum 5

VI. Magnon-photon coupling 5

VII. Alignment procedure 6

VIII. Niobium resonators in magnetic fields 6

IX. Insulating barrier devices 7

References 9

I. TECHNICAL DETAILS OF THE CALCULATION OF THE SUPERFLUID DENSITY

We now discuss the technical details of calculating the superfluid density in the simplified phenomenological model. The clean-limit of this theory agrees with the experimental results presented in the main text, while the dirty limit of the theory leads to different behavior. This limit, studied using the Usadel equation, will be presented elsewhere.

We begin by discussing the generation of the nodal p-wave condensate component inside the ferromagnet. We note that while a detailed calculation can be found in [1], here we only study a simplified model described by the 2x2 Bogolyubov-de-Gennes Hamiltonian:

$$H_{\rm BdG} = \begin{pmatrix} \xi^{(\rm s)}_{\mathbf{k}}\hat{\tau}_3 + \Delta\hat{\tau}_2\hat{\sigma}_2 & t\hat{\tau}_3 + t_{\rm soc}\left(\mathbf{k}_x\hat{\sigma}_2\hat{\tau}_3 - \mathbf{k}_y\hat{\sigma}_1\right) \\ t\hat{\tau}_3 + t_{\rm soc}\left(\mathbf{k}_x\hat{\sigma}_2\hat{\tau}_3 - \mathbf{k}_y\hat{\sigma}_1\right) & \xi^{(\rm f)}_{\mathbf{k}}\hat{\tau}_3 + h_x\hat{\tau}_3\hat{\sigma}_1 \end{pmatrix},$$

where $\xi^{(\rm s/f)}_{\mathbf{k}}$ are the electronic dispersions, h_x is the Zeeman field value, Δ is the bulk gap inside the superconductor, t is the regular tunneling and $t_{\rm soc}$ is the tunneling with spin-orbit interaction. The Nambu and spin Pauli matrices are denoted as $\hat{\tau}_i$ and $\hat{\sigma}_i$. Using the projector formalism, we now integrate out the superconductor and the minority-spin component degrees of freedom. The induced imaginary-frequency self-energy reads:

$$\hat{\Sigma}_{\mathbf{k}}\left(i\epsilon_n\right) = -\mathcal{P}H_{\rm BdG}\left(i\epsilon_n - QH_{\rm BdG}Q\right)^{-1}H_{\rm BdG}\mathcal{P}$$

where $\mathcal{P}$ is the projector onto the majority-spin component, $\mathcal{Q} = \mathbb{I} - \mathcal{P}$ and $\epsilon_n = (2n+1)\pi/\beta$. The resulting expression can be found analytically by expanding up to the second order in the tunneling but it is still too cumbersome to be reproduced here. Importantly, depending on the Fermi surface geometry and scattering properties, there are two kinds of terms: induced spin-orbit interaction $\sim \frac{2k_y t t_{\rm soc}\hat{\tau}_0}{\Delta^2+\delta E^2}\delta E$ and the nodal p-wave triplet component $\sim \frac{2k_x t t_{\rm soc}\Delta}{\Delta^2+\delta E^2}\hat{\tau}_2$, where δE is the difference of Fermi energies of the majority-spin and the superconductor. In the following, we focus only on the triplet component assuming $\hat{\Sigma}_{\mathbf{k}} \approx k_x\Delta_t\hat{\tau}_2$ with Δ_t being a free parameter.

A. Self-energy and vertex corrections

We now consider the disorder averaging and vertex corrections to the superfluid density. Within the self-consistent T-matrix approximation, the self-energy due to disorder scattering reads [2]:

$$\hat{\Sigma}\left(i\epsilon_n\right) = n_i\hat{T}\left(i\epsilon_n\right),$$

where n_i is the impurity concentration. The $\hat{T}$-matrix is given by the sum of ladder diagrams and is equal to:

$$\hat{T}\left(i\epsilon_n\right) = v_0\left(1 - v_0\left\langle\hat{G}_{\mathbf{k}}\left(i\epsilon_n\right)\right\rangle\right)^{-1},$$

where $\langle\ldots\rangle = L^{-2}\sum_k$ and v_0 is the disorder scattering strength and $G_{\mathbf{k}}$ is the full Green's function. In the main text, we take the limit $v_0 \to \infty$ and denote the scattering rate as $\tau^{-1} = n_i/\nu_0$, where ν_0 is the electronic density of states. In this case, the T-matrix is $\hat{T}\left(i\epsilon_n\right) = T\left(i\epsilon_n\right)\hat{\tau}_3$, and the remaining equation for $T(i\epsilon_n)$ can be solved self-consistently to find the self-energy.

We also need to consider the proper vertex corrections to compute the Meissner response. Within the self-consistent T-matrix approximation [2] the corrections to the current vertex Γ_μ are given by the Bethe-Salpeter equation:

$$\Gamma_\mu\left(i\epsilon_n, i\epsilon_n + i\Omega_m, \mathbf{k}\right) = \gamma_\mu\left(\mathbf{k}\right) + n_i\int\frac{d^2\mathbf{k}'}{(2\pi)^2}T\left(i\epsilon_n\right)T\left(i\epsilon_n + i\Omega_m\right)\tau_3\hat{G}_{\mathbf{k}'}\left(i\epsilon_n + i\Omega_m\right)\Gamma_\mu(i\epsilon_n, i\epsilon_n + i\Omega_m, \mathbf{k})\hat{G}_{\mathbf{k}'}(i\epsilon_n)\tau_3.$$

Following [2], we first analytically continue this equation to real frequencies and then solve it numerically.

II. QUASICLASSICAL APPROACH

We now study the SF bilayer structure using the quasiclassical Green's function approach. In this context, we find a different scaling of the superfluid order parameter compared to our toy model in the main text. We believe this difference is due to the limitations of the method, specifically its intrinsic restriction to the physics at length-scales larger than atomic scales. However, the quasiclassical approach does allow us to consider a realistic geometry where both materials can have finite thickness. Provided the proximity effect is short-range, we can assume that both materials are infinitely thick thereby occupying the left and right half-spaces. The geometry we consider is shown in Fig. 1 (a). In order to describe the proximity effect we define the Green's function in the whole space as $\check{G}(\mathbf{r},\mathbf{r}',\epsilon_n) = -\int e^{i\epsilon_n\tau}\left\langle T\Psi(\mathbf{r}',\tau)\otimes\Psi^\dagger(\mathbf{r}',0)\right\rangle$, where Ψ is the fermionic Nambu spinor. We now follow the conventional quasiclassical approach and first transform the full Green's function into momentum space with respect to the relative coordinate $\mathbf{r} = \mathbf{r} - \mathbf{r}'$ and then integrate over the magnitude of the relative momentum as follows [3]:

$$\check{g}(\mathbf{v_F},\mathbf{r}_{\rm com}) = \frac{i}{\pi}\int d\xi_k \check{G}(\xi_k,\mathbf{v}_F,\epsilon_n,\mathbf{r}_{\rm com}),$$

where $\mathbf{v}_F$ denotes the direction of the relative momentum, $\mathbf{r}_{\rm com}$ is the center-of-mass coordinate (in the following we will omit the subscript for brevity), and $\epsilon_n = \frac{2n+1}{\beta}\pi$ denotes the Matsubara frequency. In the following we denote the superconductor and ferromagnet Green's functions as follows $\check{g}_S \equiv \check{g}_S(z<0)$, $\check{g}_F \equiv \check{g}(z>0)$ and the relevant equation reads:

$$\mathbf{v}_F\cdot\nabla\check{g}_S = \left[\check{g}_S, \epsilon_n\tau_3 + \check{\Delta} + \frac{1}{2\tau_S}\langle\check{g}_S\rangle\right], \quad (1)$$

where the s-wave order parameter is defined as $\hat{\Delta} = \Delta\tau_2\sigma_2$. Inside the ferromagnet the equation is[4]:

$$\mathbf{v}_F\cdot\nabla\check{g}_F = \left[\check{g}_F, \epsilon_n\tau_3 + ih_x\sigma_1 + \frac{1}{2\tau_F}\langle\check{g}_F\rangle\right], \quad (2)$$

where we assumed the Zeeman field h is oriented along the x direction as shown in Fig. 1 (a). Since the system is translationally invariant in the x and y directions, the Green's function depends only on z and therefore $\mathbf{v}_F\cdot\nabla = v_{F,x}\partial_z \equiv v_F\mu\partial_z$, where we denoted $\mu \equiv v_{F,x}/v_F$. We included the disorder scattering in Eqs. (1,2) and τ_S, τ_F denote the disorder scattering time in the superconductor and ferromagnet respectively. $\langle\ldots\rangle$ denotes an angular average over $\mathbf{v}_F$. Finally, in order to determine the quasiclassical Green's function throughout the whole system, the boundary condition (BC) needs to be provided. In the following we will use two types of BC: transparent and weak tunneling. Transparent boundary conditions imply that the Green's function is simply continuous at the interface. In the regime of weak tunneling, the boundary condition is derived in [5]. Here we will only be interested in the ferromagnet Green's function obeying for $\mu > 0$:

$$\check{g}_F(\mu,0) - \check{S}_{FF}\check{g}_F(-\mu,0)\check{S}_{FF}^{-1} = -\frac{1}{2}\left[\check{S}_{FS}\check{g}_S(\mu)\check{S}_{SF}^\dagger, \check{g}_F(\mu,0)\right], \quad (3)$$

where S_{ij} with $i,j = S/F$ denotes different components of the interface scattering matrix. We note that additional terms like the external gauge field coupling straightforwardly added to Eqs. (1, 2) as $-i\mathbf{v}_F\mathbf{A}\tau_3$. Finally, the quasiclassical Green's function allows for the evaluation of different observables such as a supercurrent density which can be expressed as [3]:

$$\mathbf{j} = -2i\pi\nu_0\frac{1}{\beta}\sum_{\epsilon_n}\langle\mathbf{v}_F\tau_3\check{g}\rangle, \quad (4)$$

where ν_0 is the fermionic density of states at the Fermi level. Remarkably, Eq. 4 incorporates both dia- and paramagnetic contributions [6].

A. Numerical solution

Despite its seeming simplicity, the numerical solution equations Eqs. (1, 2) is a complicated problem due to the presence of divergent unphysical solutions. The existing methods which allow for the elimination of such solutions include the so-called "explosion method" [7] and the method of Riccati parametrization explained in detail in [8]. In this work we numerically implement the Riccati method which allows for an efficient solution. The result of numerical simulation is shown in Fig. 1 (b). We observe generation of different components including a p-wave triplet with the nodes of the superconducting gap oriented transverse to the Zeeman field. We provide the intuition on the propagation and generation of different components in the next section. Here we now estimate the temperature dependence of the superfluid density which we derive by adding an external uniform gauge field as discussed above. We then integrate the resulting current density along the z direction inside the ferromagnet, defining $\mathbf{I}(T,\mathbf{A}) = \int_{z>0} dz\mathbf{j}$. The scaling of different components of $\mathbf{I}$ with temperature provides $\delta K_{ii}/\delta K_{ii}(0) = \mathbf{I}_i(T,\mathbf{A})/\mathbf{I}_i(T=0,\mathbf{A})$. The result is shown in Fig. 1 (c) and we observe a T^2 scaling in both directions.

B. Generation and propagation of p-wave triplet

In this section, we provide a general description of the propagation of the p-wave component induced inside the

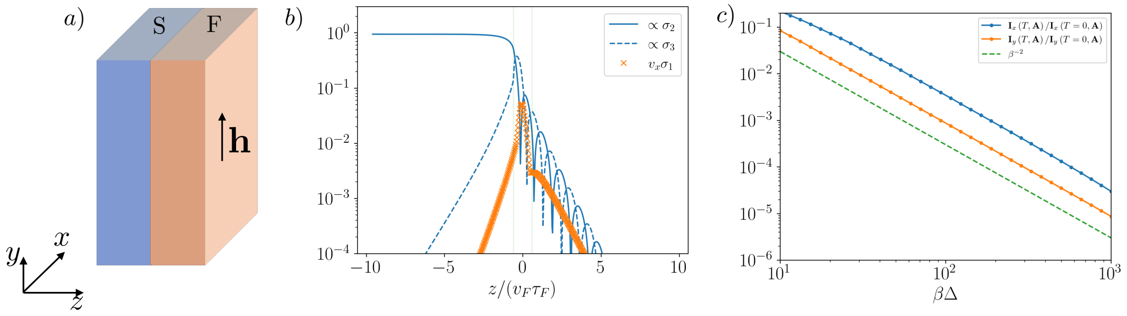

FIG. 1. Proximity effect in a superconductor-ferromagnet heterostructure. a) schematic of the model system, b) superconducting correlations induced inside ferromagnet. c) superfluid density as function of temperature. Parameters of the simulation: $h_x = 5\Delta, \Delta\tau_{S/F} = 1/6$. The Rashba spin-orbit is confined to a small interface region $\pm v_F\tau_{S/F}$ and its value is taken to be $v_F t_{\rm soc} = 5\Delta$.

ferromagnet. To make the problem tractable, we assume that the interface is weakly transmissive and comment on the generation of the p-wave triplet in a highly transmissive interface at the end. Let us first demonstrate the generation of the p-wave triplet due to spin-orbit scattering. We now use the following expression for the interface scattering, which encodes the spin-orbit tunneling (here, we only provide the particle-particle part, while the hole-hole part can be straightforwardly obtained [5]): $\tilde{S} = S^{\rm tr}\left(-\mathbf{p}_{\parallel}\right)$. The particle-hole part of the scattering matrix is assumed to be zero [5] for simplicity.

$$\hat{S}_{SF} = it\hat{\mathbb{I}}_2 - it_{\rm soc}\left[\mathbf{v}_{\parallel} \times \mathbf{s}\right]_z$$
$$\hat{S}_{FF} = -\hat{\mathbb{I}}_2\left(1 - it\right) - it_{\rm soc}\left[\mathbf{v}_{\parallel} \times \mathbf{s}\right]_z$$

Where $\mathbf{v}_{\parallel}$ is the in-plane projection of the Fermi velocity and the spin operator is denoted as $\mathbf{s} = \{\sigma_1, \sigma_2\tau_3, \sigma_3\}$, $t_{\rm soc}$ and t denote the tunneling coefficients with and without the spin-orbit coupling respectively. In the limit $t, t_{\rm soc} = 0$ the Green's functions are space independent and are given by:

$$\check{g}_S^{(0)} = \frac{\epsilon_n\tau_3\sigma_0 + \Delta\tau_2\sigma_2}{\sqrt{\epsilon_n^2 + \Delta^2}}, \check{g}_F^{(0)} = \mathrm{sign}(\epsilon_n)\tau_3\sigma_0.$$

With this we find

$$S_{FS}\tau_2\sigma_2 S_{SF}^{\dagger} = \left(it\hat{\mathbb{I}}_4 - it_{\rm soc}\left[v_x\sigma_2 - v_y\sigma_1\tau_3\right]\right)\tau_2\sigma_2\left(-it\hat{\mathbb{I}}_4 + it_{\rm soc}\left[v_x\sigma_2 - v_y\sigma_1\tau_3\right]\right)$$
$$\approx \left(t^2\tau_2\sigma_2 - 2tt_{\rm soc}\left\{v_x\tau_2 - v_y\tau_1\sigma_3\right\}\right)$$

And finally to lowest order in tunneling we get:

$$-\frac{1}{2}\left[S_{FS}\left(\check{g}_S^{(0)}\right)S_{SF}^{\dagger}, \check{g}_F^{(0)}\left(0\right)\right] = -\frac{1}{2}\left[\left(\frac{\Delta\left(t^2\tau_2\sigma_2 - 2tt_{\rm soc}\left\{v_x\tau_2 - v_y\tau_1\sigma_3\right\}\right)}{\sqrt{\epsilon_n^2 + \Delta^2}}\right), \mathrm{sign}(\epsilon_n)\tau_3\right]$$
$$- i\,\mathrm{sign}(\epsilon_n)\frac{\Delta}{\sqrt{\epsilon_n^2 + \Delta^2}}\left(t^2\tau_1\sigma_2 - 2tt_{\rm soc}\left\{v_x\tau_1 + v_y\tau_2\sigma_3\right\}\right) \quad (5)$$

We thus find the generation of several different superconducting correlations including a spin-singlet ($\propto \sigma_2$ in our notations) and two different p-wave triplet components ($\propto \sigma_0$ and $\propto \sigma_3$ respectively). Assuming a weakly transparent interface, we linearize Eq. (2) using the ansatz $\check{g}_F = \check{g}_F^{(0)} + \check{f}$, where $\check{f}$ is purely off-diagonal in Nambu space:

$$\mu\mathrm{sign}\left(\epsilon_n\right)\tau_3\partial_{\tilde{z}}\check{f} = -\kappa_n\check{f} + i\tau_F\mathrm{sign}\left(\epsilon_n\right)h_x\tau_3\left[\check{f}, \sigma_1\right] + \langle\check{f}_F\rangle.$$

where $\tilde{z} = z/v_F\tau_F$ and $\kappa_n = \left(1 + 2\left|\epsilon_n\right|\tau_F\right)$. We now define the symmetric and antisymmetric components as $\check{a} \equiv \check{f}\left(\mu\right) - \check{f}\left(-\mu\right)$ and $\check{s} \equiv \check{f}\left(\mu\right) + \check{f}\left(-\mu\right)$

$$\mu \mathrm{sign}\left(\epsilon_n\right)\tau_3\partial_{\tilde{z}}\check{s} = -\kappa_n\check{a} + i\tau_F \mathrm{sign}\left(\epsilon_n\right) h_x\tau_3\left[\check{a},\sigma_1\right] \quad (6)$$

$$\mu \mathrm{sign}\left(\epsilon_n\right)\tau_3\partial_{\tilde{z}}\check{a} = -\kappa_n\check{s} + i\tau_F \mathrm{sign}\left(\epsilon_n\right) h_x\tau_3\left[\check{s},\sigma_1\right] + \langle\check{s}\rangle \quad (7)$$

with the boundary condition (Eqs. 3, 5):

$$\check{a}\left(\tilde{z}=0\right) = -i\mathrm{sign}(\epsilon_n)\mathrm{sign}\left(\mu\right)\frac{\Delta}{\sqrt{\epsilon_n^2+\Delta^2}}\left(t^2\tau_1\sigma_2 - 2tt_{\mathrm{soc}}\left\{v_x\tau_1 + v_y\tau_2\sigma_3\right\}\right) \quad (8)$$

Motivated by the band structure of permalloy, particularly the difference in densities of states and Fermi velocities of the two spin components, we will effectively project onto the majority spin component in the following discussion. This component is encoded in the $\tau_1\sigma_2$ term of the boundary condition Eq. (5). By using the ansatz $\check{s} = \sigma_0\hat{s}_0, \check{a}_0 = \sigma_0\hat{a}_0$, and considering that p-wave is odd in momentum ($\langle\hat{s}_0\rangle = 0$), we solve Eqs.(6), (7) and get:

$$\hat{s}_0\left(k\right) = \frac{-2\left|\mu\right|\kappa_n}{k^2\mu^2+\kappa_n^2}\frac{\Delta 2tt_{\mathrm{soc}}}{\sqrt{\epsilon_n^2+\Delta^2}}v_x\tau_2 \quad (9)$$

We thus find that the triplet component is short-range in diffusive limit ($\kappa_n \approx 1$) with the effective penetration range of the order of mean free path as can be seen from the denominator. We note that the projection of the pair spin onto the Zeeman axis is $S_x = \pm 1$ which explains the absence of oscillations in space. The same is also true for the transparent interface with the only difference being the phase between the $S_x = 1$ and $S_x = -1$ (the condensate has spin structure σ_1 in contrast to σ_0 in Eq. 9). Let us now clarify the generation of the triplet at transparent interface. The Eilenberger equations Eqs. ((6), (7)) in the presence of spin-orbit and Zeeman are modified as follows:

$$\begin{aligned}\mathrm{sign}\epsilon_n\mu\tau_3\partial_{\tilde{z}}\check{s} = &-\kappa_n\check{a} + \tau_F\mathrm{sign}\epsilon_n i h_x\tau_3\left[\check{a},\sigma_1\right]\\ &+ \tau_F\mathrm{sign}\epsilon_n i t_{\mathrm{soc}}v_x\tau_3\left[\check{a},\sigma_2\right]\\ &+ \tau_F\mathrm{sign}\epsilon_n i t_{\mathrm{soc}}v_y\left\{\check{a},\sigma_1\right\} \quad (10)\end{aligned}$$

$$\begin{aligned}\mathrm{sign}\epsilon_n\mu\tau_3\partial_{\tilde{z}}\check{a} = &-\kappa_n\check{a} + \tau\mathrm{sign}\epsilon_n i h_x\tau_3\left[\check{s},\sigma_1\right] + \langle\check{s}\rangle\\ &+ \tau_F\mathrm{sign}\epsilon_n i t_{\mathrm{soc}}v_x\tau_3\left[\check{s},\sigma_2\right]\\ &+ \tau_F\mathrm{sign}\epsilon_n i t_{\mathrm{soc}}v_y\left\{\check{s},\sigma_1\right\} \quad (11)\end{aligned}$$

Generation of the triplet is now achieved in two steps: first the isotropic s-wave component $\tau_2\sigma_2$ undergoes a precession in Zeeman field inducing, in particular, an antisymmetric component $\mu\check{a}_F \propto \tau_2\sigma_3$ (we retain μ here in order to indicate the proper Fermionic symmetry of this component). According to Eq. (10) this component then then be transformed into a p-wave triplet as follows $\check{s}_F \propto v_x\tau_2\sigma_1$. Outside the region with spin-orbit coupling this component has the same equation as $\tau_2\sigma_0$.

III. DEVICE FABRICATION

The devices studied in this work are fabricated by first thermally evaporating gold bond pads and alignment marks onto a high-resistivity intrinsic silicon substrate. The chip is dipped in hydroflouric (HF) acid and a 25 nm thick Nb film is immediately sputtered onto the cleaned chip. The resonator structure is defined using electron-beam lithography and the unwanted Nb is removed via reactive ion etching with CF_4. To fabricate the S/F hybrid resonators, the S/F bilayer region at the end of the resonator is defined in another electron-beam lithography step. The exposed Nb region is again cleaned in HF to ensure a transparent interface, after which a 30 nm thick permalloy film is immediately thermally evaporated.

IV. EXPERIMENTAL SETUP

The experiments described in this work are performed in a dilution refrigerator (Oxford Instruments Kelvinox MX50) with a base temperature of 55 mK equipped with a three-axis vector magnet. Microwave signals generated by a vector network analyzer (Keysight PNA Microwave Network Analyzer N5227B) are sent down a stainless steel coaxial line which is thermally anchored via attenuators to each plate of the cryostat as illustrated in Fig. 2a. The sample is mounted on the mixing chamber in a copper sample holder designed by IBM Research, and is connected to the measurement circuit via non-superconducting gold wirebonds. The signal transmitted across the device is routed through a circulator (Quinstar QCY-G0400801) via Nb superconducting coaxial lines to a cold amplifier (Low Noise Factory LNF-LNC03-14SA) on the 4K plate. The amplified signal then leaves the cryostat via stainless steel coaxial lines and is further amplified at room temperature (MITEQ LNA-40-04000800-07-10P) before being read out into one of the ports of the network analyzer.

The microwave transmission S_{21} is recorded and fit to the standard form for a hanging resonator [9] to extract the resonance frequency. Representative traces of S_{21} versus frequency featuring the resonator mode for devices terminated both with and without the S/F bilayer are shown in Fig. 2b, along with the corresponding fits used

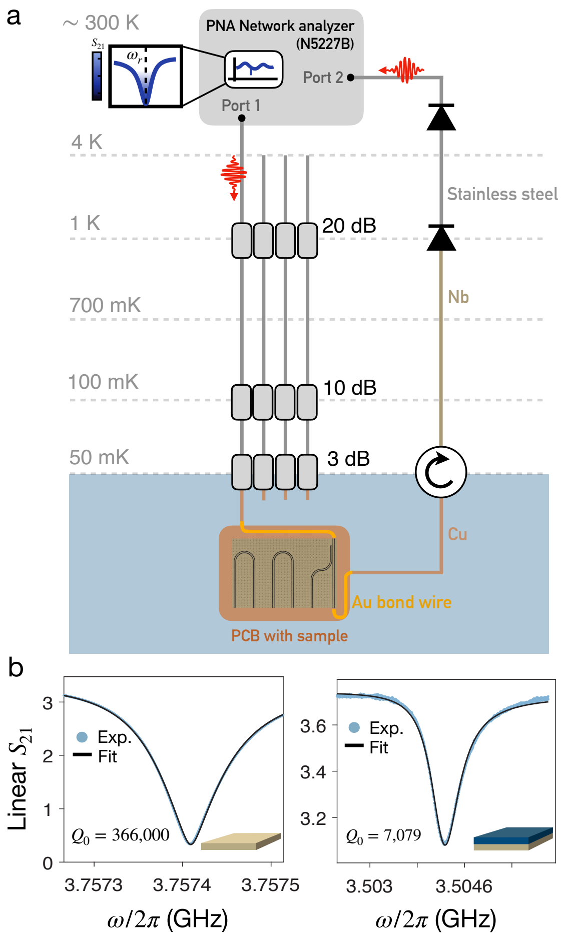

FIG. 2. **Experimental setup**. **a.** Schematic wiring diagram for the microwave measurement setup. All lines are coaxial cables, with the materials for each segment indicated in the figure. Grey boxes represent attenuators thermally anchored to each plate of the dilution refrigerator. **b.** Traces of the microwave transmission S_{21} for bare Nb resonators and hybrid resonators terminated with an S/F bilayer, respectively. The fit used to extract the resonance frequency is shown along with the raw data, along with the internal quality factor obtained from the fit.

to extract the resonance frequency.

V. MEASURING THE SUPERCONDUCTING GAP OF ALUMINUM

As a proof of concept for our kinetic inductance measurement technique, we studied a device in which the Nb resonator is shorted to ground through a small Al strip, rather than an S/F bilayer. The termination of the resonator is shown in Fig. 3a: the center conductor of the Nb resonator is “cut” and replaced with a 40 μm long, 20 nm thick Al film with a width of either 2.5 μm or 5 μm. As discussed in the main text, the resonator design localizes most of the current to the Al strip, making the resonator response particularly sensitive to the Al region. Moreover, since the critical temperature and superconducting gap of Al are much lower than that of Nb, the temperature dependence of the resonator frequency will be almost exclusively due to the temperature dependence of the superfluid density in the Al strip. In Fig. 3b,c we show the temperature dependence of the resonance frequency for the two resonators with different widths of the Al strips. In both cases, the curves are activated with temperature, as one would expect for a fully-gapped conventional superconductor. Moreover, we may fit these data to the low-temperature limit of the standard BCS form for the superfluid density [10],

$$\frac{\delta f}{f_0} = A\sqrt{\frac{2\pi\Delta_0}{T}}\,\mathrm{e}^{-\Delta_0/T}\,. \tag{12}$$

The amplitude of the frequency shift, A, and the zero-temperature superconducting gap Δ_0 are treated as fit parameters. The fits to each curve are superimposed on the data in Fig. 3b,c, and yield values of the gap $\Delta_0 = 240$ μeV (280 μeV) for the resonator with a 2.5 μm (5 μm) Al strip. These values are consistent with direct measurements of the superconducting gap of Al thin films, which quantitatively validates our measurement technique and analysis procedure.

VI. MAGNON-PHOTON COUPLING

As described in the main text, by tuning an external field such that the Kittel mode frequency, $\omega_m = \gamma\sqrt{\mu_0^2 H_\parallel(H_\parallel + M_s)}$, coincides with that of the resonator, ω_r, avoided crossings are observed symmetrically around zero for the first and third harmonic of the resonator. When the magnons couple to the photons, the hybrid mode is highly broadened due to magnon damping. We obtain the magnon-photon coupling strength, g, from modeling the two bands, using the following equation for the transmission spectrum [11–13]

$$S_{21}(\omega, H_\parallel) = \frac{\kappa_{r,ext}}{i(\omega-\omega_r) - \kappa_r + \frac{g^2}{i[\omega-\omega_m(H_\parallel)]-\kappa_m/2}} \tag{13}$$

where κ_r and $\kappa_{r,ext}$ are the resonator internal and external loss rate respectively and κ_m is the magnon damping rate. From this we determine the total magnon-photon coupling to be $g_{1\mathrm{st}} = 120$ MHz for the first harmonic, consistent with the coupling extracted from anticrossing of the third harmonic of the resonator, $g_{3\mathrm{rd}} = 100$ MHz. We note that the extracted saturation magnetization of the first mode, $M_s^{1\mathrm{st}}$ is more than twice the value that we obtain from the third harmonic, $\mu_0 M_s^{3\mathrm{rd}} = 1.38$ T, and what has previously been reported in the literature [12, 13]. We speculate that trapped fields could lead to a seemingly larger saturation magnetization.

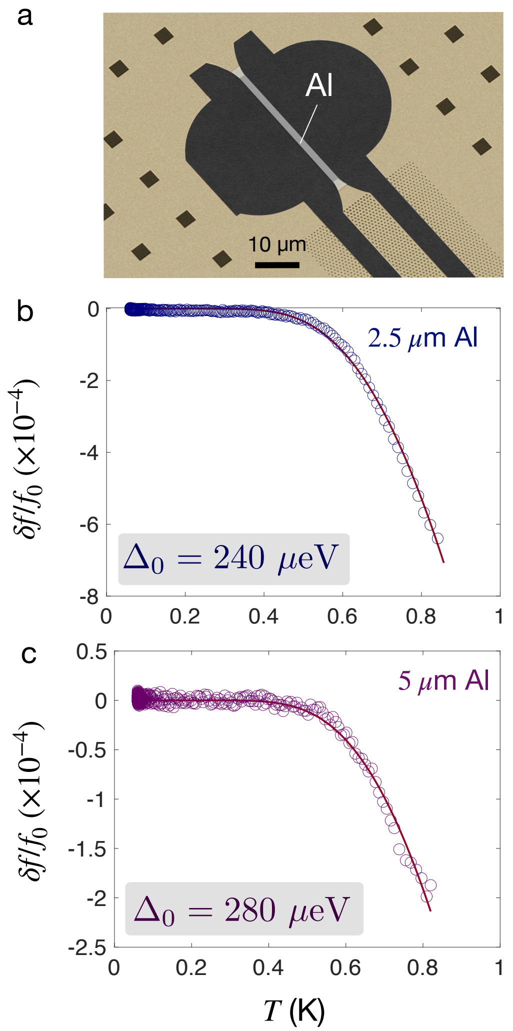

FIG. 3. **Aluminum hybrid resonators**. **a.** False-colored scanning electron micrograph of the Al strip terminating a Nb resonator otherwise identical to that used in the S/F bilayer devices, as described at length in the main text. **b.** Temperature-dependent resonance frequency of a device with a 2.5 μm wide Al strip. The data is fit to Eq. (12), which yields a value of $\Delta_0 = 240\ \mu$eV for the superconducting gap. **c.** Temperature-dependent resonance frequency and BCS fit for a device with a 5 μm wide Al strip.

VII. ALIGNMENT PROCEDURE

The measurements described in the main text are performed in an in-plane magnetic field, although in reality sample misalignment inevitably leads to small out-of-plane field components. To eliminate the effects of these small unwanted out-of-plane fields, we employ the three-axis vector magnet in our cryostat to compensate for the out-of-plane field and ensure that the magnetic field experienced by the sample is entirely in-plane. To do so, we use the resonator frequency as a sensitive measure of the field experienced by the sample. The resonance frequency of the superconducting resonator decreases with applied out-of-plane fields in an approximately parabolic fashion for small fields due to field-induced pair breaking and the associated decrease in superfluid density (or, equivalently, increase in kinetic inductance), as shown in Fig. 4a. The maximum of this parabola indicates the "effective" zero-field where the out-of-plane field experienced by the sample vanishes.

To align the magnetic field, we begin by setting the nominal in-plane field to its desired value. At this fixed in-plane field, we sweep the out-of-plane field and determine the applied out-of-plane field corresponding to the effective zero-field as described above. We then retrace the out-of-plane field history to avoid any hysteretic effects and set the out-of-plane field to it's effective-zero-field value (i.e. the applied field corresponding to the maximum of the parabola). With this field configuration fixed, we then proceed with our temperature-dependent scans. Similar techniques for field alignment have been employed in previous studies of superconducting resonators [14]. We also note that the temperature-dependent sweeps will occasionally experience stochastic "jumps" where the frequency abruptly jumps by several MHz. These large, easily identifiable jumps are subtracted out of the raw data, resulting in the smooth traces presented in the main text.

If this alignment procedure is not followed, temperature-dependent resonance frequency traces often feature artifacts due to trapped vortices. An example of one such effect, a pronounced "downturn" in the resonance frequency as the temperature is lowered, is shown in Fig. 4b. These artifacts are strongly history-dependent and non-systematic. In contrast, when the alignment procedure described above is followed (as is the case for all data presented throughout the main text), the temperature-dependent traces are free of these artifacts and are highly reproducible.

VIII. NIOBIUM RESONATORS IN MAGNETIC FIELDS

In this section we discuss the phenomenology of bare Nb resonators (i.e. without S/F bilayers) subject to magnetic fields, and contrast their behavior to that of the hybrid S/F resonators studied in the main text. In Fig. 5a we show temperature-dependent traces of the resonant frequency for a Nb resonator subject to an in-plane field of $\mu_0 H_\parallel = 20$ mT for varying values of the out-of-plane magnetic field.

We find that the temperature dependence of the frequency is well described by the BCS activated dependence in contrast to a power law fitting as we obtained in the main text. In addition, we find that at the lowest temperature we find a small 'downturn', which has been well described in the literature as a consequence of coupling to a nearby TLS. Our fit function in addition to the BCS contribution, takes the TLS contribution into account with a single fit parameter, Q_{TLS}, the quality

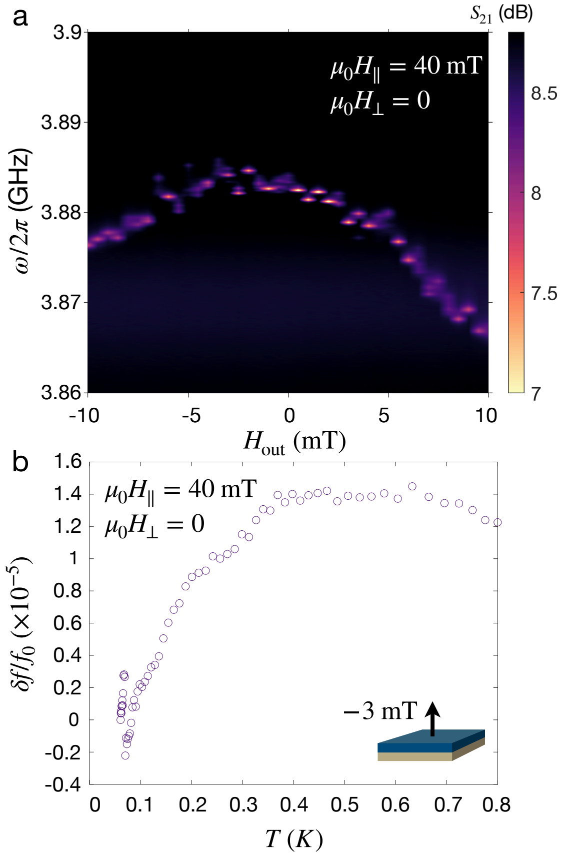

FIG. 4. **Field alignment procedure**. **a.** Resonance frequency of a S/F hybrid resonator as a function of the applied out-of-plane magnetic field. The maximum frequency of the resonator corresponds to the effective zero-field. **b.** Example of a temperature scan of the resonance frequency when the field alignment procedure is not followed and the temperature-dependence exhibits non-systematic behavior.

factor associated with the TLS [15]. The full fit function, including both BCS (equation 12) and TLS contributions, is

$$\frac{\delta f(T)}{f_0} = \left(\frac{\delta f(T)}{f_0}\right)_{\rm BCS} + \left(\frac{\delta f(T)}{f_0}\right)_{\rm TLS}$$

with

$$\left(\frac{\delta f(T)}{f_0}\right)_{\rm TLS} = \frac{1}{\pi Q_{\rm TLS,0}} {\rm Re}\left[\psi\left(\frac{1}{2} + i\frac{\hbar\omega}{2\pi k_B T}\right) - \ln\left(\frac{\hbar\omega}{2\pi k_B T}\right)\right],$$

where $\psi(z)$ is the digamma function. In general, we find the TLS contribution is rather weak, and is only sometimes observed at a few particular field values. Fig. 5a plots the trace with a small out-of-plane field of 3 mT, with no apparent TLS contribution (no downturn at lowest temperature), contrasted to Fig. 5b, where the applied field is 5 mT. To highlight TLS contribution, i.e. the deviation from an activated temperature dependence, we have presented an Arrhenius plot with $\log {\rm abs}(\delta f/f_0)$ plotted as a function of T^{-1}. Other than stochastic coupling to TLS with applied out-of-plane field, the scaling with temperature is otherwise always well described with activation and the only systematic change is the overall magnitude of the change. To illustrate this effect, we introduce the total frequency shift $S = [f(55 \text{ mK}) - f(800 \text{ mK})]/f(55 \text{ mK})$ which quantifies the overall size of the frequency shift with temperature in each run. We plot the extracted S and n as a function of the out-of-plane field in Fig. 5c,d, where we see that the temperature-scaling exponent is unchanged by the out-of-plane field. In contrast, the net frequency shift S increases monotonically with the out-of-plane field, presumably due to the reduction of the superfluid density and commensurate decrease of f_0. To emphasize the insensitivity of the temperature scaling to magnetic fields, in Fig. 5b we normalize the frequency shifts to S, and see that the curves for each out-of-plane field collapse onto one another. Thus, the sole effect of the out-of-plane magnetic field is to rescale the total size of the frequency shift, and does not affect the temperature scaling in any way. We also emphasize that in all cases, the overall magnitude of the frequency shift observed in the bare resonators is an order of magnitude smaller than the shifts observed in the hybrid S/F devices studied in the main text.

Further, in Fig. 5e we compare temperature-dependent traces in in-plane fields along both orthogonal directions $H_{\|}$ and $H_{\perp}$ studied in the main text. We note that in this particular cooldown, the misalignment in the $H_{\perp}$ direction was somewhat large, with $H_{\rm out} \approx 20$ mT corresponding to the effective zero field. Again, independent of the out-of-plane magnetic field, all curves display an activated temperature dependence. Normalizing by S as before, we find that the temperature-dependent traces for both in-plane field directions collapse onto one another. That is, the temperature dependent response of bare Nb resonators is activated irrespective of applied magnetic fields and isotropic with respect to the orientation of in-plane fields.

IX. INSULATING BARRIER DEVICES

In addition to the devices featuring a direct superconductor/ferromagnet interface discussed in the main text, we also fabricated and measured devices where the ferromagnetic and superconducting layers are electrically isolated by an insulating dielectric layer. In this geometry, the insulating barrier prevents any proximity-coupling between the superconductor and ferromagnet, and enables us to isolate and study any possible effects of stray dipolar fields from the ferromagnet on the resonator response.

These devices were fabricated by first depositing and

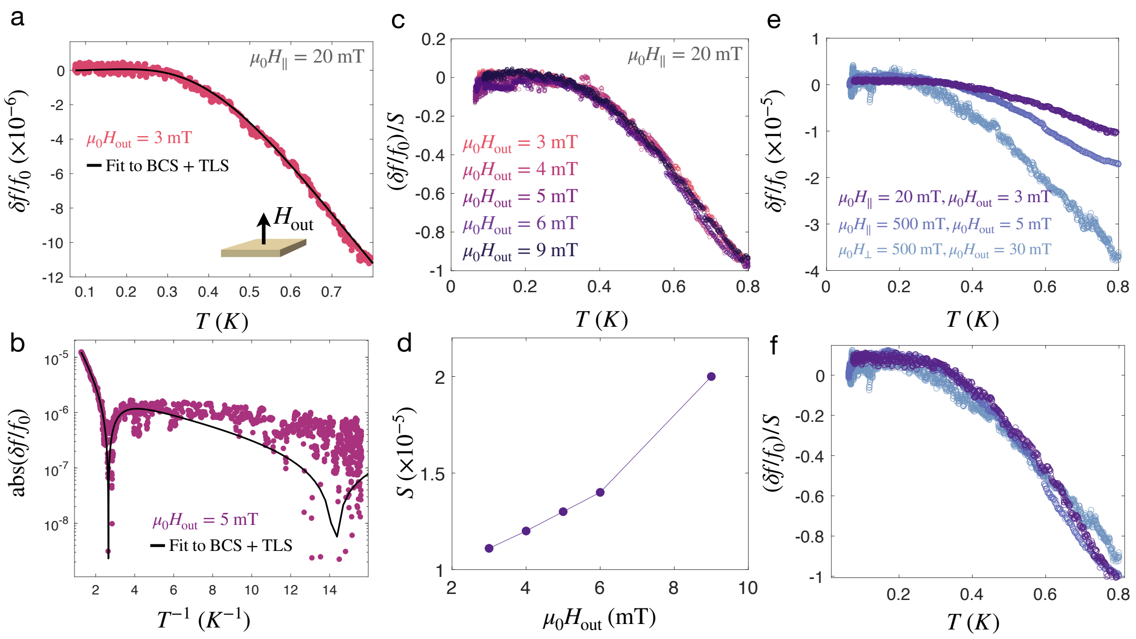

FIG. 5. **Niobium resonators in magnetic fields**. **a.** Fit to the temperature-dependent resonance frequency of a Nb resonator subject to an in-plane magnetic field $\mu_0 H_{\parallel} = 20mT$ for one out-of-plane field (3 mT), exhibit activated temperature dependene with a small contribution from TLS at lowest temperature. **b.** Arrhenius plot of the temperature-dependent resonance frequency at a different out-of-plane field (5 mT), here the TLS contribution is present at lowest temperature. **c.** Data in panel **a** normalized to the integrated frequency shift S. **d.** Extracted net frequency shift S. **e** Temperature-dependent resonance frequency for Nb resonators subjected to in-plane magnetic fields in both the $H_{\parallel}$ and $H_{\perp}$ directions. **e.** Data from panel **d** normalized to the total frequency shift S.

patterning the Nb resonator as described above, then defining the superconductor/insulator/ferromagnet heterostructure with electron-beam lithography followed by sputtering an amorphous oxide layer and subsequently thermally evaporating the permalloy film. As shown below, we have studied devices with both SiO_2 and MgO insulating barriers. In both cases, these amorphous oxides present a much dirtier dielectric environment for the resonator compared to the pristine intrinsic Si substrate our devices are fabricated on. As a consequence, the resonator response is affected by the considerable density of two-level systems (TLS) in the oxide layers.

In Fig. 6a, we present the temperature-dependent resonance frequency of the third harmonic of the resonator with a 100 nm thick SiO2 insulating barrier between the Nb resonator and Py stripe. Training the Py moment along each of the two in-plane orientations with a 10 mT magnetic field, we see that in both cases the resonator frequency evolves slowly with temperature, with a dependence that is isotropic and an overall frequency shift is of the same order of magnitude as what we observe in bare Nb resonators at low magnetic fields. That is, these superconductor/insulator/ferromagnet devices behave more similarly to bare Nb resonators than to the superconductor/ferromagnet devices studied in the main text. We note however, that due to the hysteretic coupling to the TLS bath, one of the experimental runs exhibits a stronger coupling to TLS's, with a more pronounced non-monotonic temperature dependence at the lowest temperatures. This non-monotonic temperature dependence is characteristic of strong coupling to TLS, as has been observed elsewhere in the literature [15].

This behavior is reproduced in the second superconductor/insulator/ferromagnet device we studied with a 50 nm thick MgO barrier. To demonstrate the inherent run-to-run variability arising from the dirty dielectric environment, we present in Fig. 6b,c multiple temperature-dependent measurements of the resonance frequency of the first harmonic of this resonator in an in-plane magnetic field of 450 mT, directed along both in-plane directions. We see that the temperature dependence is again dominated by the TLS response, with a variable TLS coupling strength between different experimental runs. Within the run-to-run variability in the frequency traces, there is no systematic anisotropy in the resonator response, unlike in the devices with a direct superconductor/ferromagnet interface. The overall frequency shift observed in these devices is again comparable to that of bare Nb resonators, and almost an order of magnitude smaller than the power-law temperature dependent frequency shift exhibited by the direct interface superconductor/ferromagnet devices in similar (in fact, lower) in-plane magnetic fields (shown in Fig. 6b for comparison).

From these experiments, we find that the superconductor/insulator/ferromagnet heterostructure devices ex-

hibit a temperature-dependent response characterized by a weak overall frequency shift (of the same order as that of resonators with no ferromagnetic layer) with a non-monotonic temperature dependence characteristic of strong coupling to TLS in the oxide layer. This response exhibits no in-plane anisotropy within the inherent run-to-run variation of the TLS coupling strength. This is in stark contrast to the strong, anisotropic power-law scaling observed in the devices featuring direct superconductor/ferromagnet interfaces studied in the main text. This suggests that the anisotropic response studied in the main text cannot be attributed to simple dipolar effects (e.g. stray fields from the ferromagnet), and are a manifestation of anisotropic proximity-induced pairing in the superconductor/ferromagnet hybrid system.

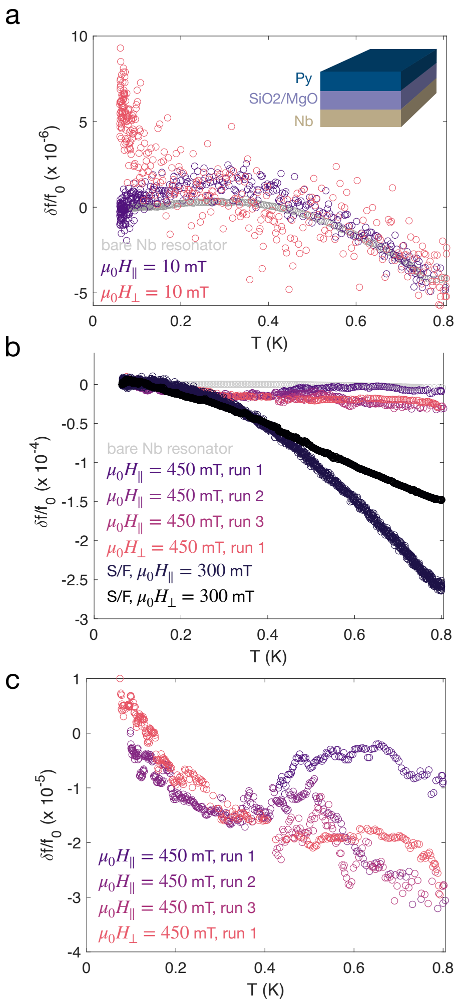

FIG. 6. **Superconductor/insulator/ferromagnet devices**. **a.** Temperature-dependent resonant frequency of the superconductor/insulator/ferromagnet device with a 100 nm thick SiO_2 insulating barrier for both orientations of in-plane magnetic field, together with temperature-dependent resonant frequency of a bare Nb resonator (grey) for comparison; inset: schematic of the superconductor/insulator/ferromagnet trilayer structure. **b.** Repeated temperature-dependent resonant frequency traces for the superconductor/insulator/ferromagnet device with a 50 nm thick MgO barrier with both in-plane field orientations. For comparison, we also plot the temperature-dependent response of a bare Nb resonator and the anisotropic power-law response observed in the direct interface superconductor/ferromagnet devices. **c.** Enhanced view of the temperature-dependent response of the MgO resonators from panel (b), where the non-monotonic TLS-dominated lineshape is apparent.

[1] S. Takei and V. Galitski, Microscopic theory for a ferromagnetic nanowire/superconductor heterostructure: Transport, fluctuations, and topological superconductivity, Physical Review B **86**, 054521 (2012).

[2] G. D. Mahan, Many-particle physics (Springer Science & Business Media, 2000).

[3] W. Belzig, F. K. Wilhelm, C. Bruder, G. Schön, and A. D. Zaikin, Quasiclassical green's function approach

to mesoscopic superconductivity, Superlattices and microstructures **25**, 1251 (1999).

[4] A. Volkov and K. Efetov, Odd triplet superconductivity in a superconductor/ferromagnet structure with a narrow domain wall, Physical Review B **78**, 024519 (2008).

[5] A. Millis, D. Rainer, and J. Sauls, Quasiclassical theory of superconductivity near magnetically active interfaces, Physical Review B **38**, 4504 (1988).

[6] N. Kopnin, Theory of Nonequilibrium Superconductivity (Oxford University Press, 2001).

[7] E. Thuneberg, J. Kurkijärvi, and D. Rainer, Elementary-flux-pinning potential in type-ii superconductors, Physical Review B **29**, 3913 (1984).

[8] N. Schopohl, Transformation of the eilenberger equations of superconductivity to a scalar riccati equation, arXiv preprint cond-mat/9804064 (1998).

[9] Q.-M. Chen, M. Pfeiffer, M. Partanen, F. Fesquet, K. E. Honasoge, F. Kronowetter, Y. Nojiri, M. Renger, K. G. Fedorov, A. Marx, F. Deppe, and R. Gross, Scattering coefficients of superconducting microwave resonators. i. transfer matrix approach, Phys. Rev. B **106**, 214505 (2022).

[10] R. Prozorov and R. W. Giannetta, Magnetic penetration depth in unconventional superconductors, Superconductor Science and Technology **19**, R41 (2006).

[11] A. Blais, A. L. Grimsmo, S. M. Girvin, and A. Wallraff, Circuit quantum electrodynamics, Rev. Mod. Phys. **93**, 025005 (2021).

[12] J. T. Hou and L. Liu, Strong coupling between microwave photons and nanomagnet magnons, Phys. Rev. Lett. **123**, 107702 (2019).

[13] Y. Li, T. Polakovic, Y.-L. Wang, J. Xu, S. Lendinez, Z. Zhang, J. Ding, T. Khaire, H. Saglam, R. Divan, J. Pearson, W.-K. Kwok, Z. Xiao, V. Novosad, A. Hoffmann, and W. Zhang, Strong coupling between magnons and microwave photons in on-chip ferromagnet-superconductor thin-film devices, Phys. Rev. Lett. **123**, 107701 (2019).

[14] J. Makita, C. Sundahl, G. Ciovati, C. B. Eom, and A. Gurevich, Nonlinear meissner effect in nb_3Sn coplanar resonators, Phys. Rev. Res. **4**, 013156 (2022).

[15] K. D. Crowley, R. A. McLellan, A. Dutta, N. Shumiya, A. P. M. Place, X. H. Le, Y. Gang, T. Madhavan, M. P. Bland, R. Chang, N. Khedkar, Y. C. Feng, E. A. Umbarkar, X. Gui, L. V. H. Rodgers, Y. Jia, M. M. Feldman, S. A. Lyon, M. Liu, R. J. Cava, A. A. Houck, and N. P. de Leon, Disentangling losses in tantalum superconducting circuits, Phys. Rev. X **13**, 041005 (2023).
